# PREVALENCE OF SCOLIOSIS IN PUBLIC ELEMENTARY SCHOOL STUDENTS

**DOI:** 10.1590/1984-0462/;2017;35;2;00008

**Published:** 2017

**Authors:** Maria Célia Cunha Ciaccia, Julia Silvestre de Castro, Mariana Abduch Rahal, Barbarah Silveira Penatti, Iara Borin Selegatto, João Lucas Morette Giampietro, Vera Esteves Vagnozzi Rullo

**Affiliations:** a Centro Universitário Lusíada de Santos, São Paulo, SP, Brasil.

**Keywords:** scoliosis, epidemiology, prevalence, education, obesity

## Abstract

**Objective:**

To evaluate the prevalence of scoliosis and the risk factors in elementary school students.

**Methods:**

A cross-sectional study was carried out in 954 students in 2015. The instrument involved was a questionnaire on postural habits, socioeconomic conditions, and demographic factors. The anthropometric measurements, including height and weight, a visual inspection of the deformity of the vertebral column, the leveling of shoulders, and the Adam’s test were obtained. The sample was calculated in the expected frequency of 12.3%, acceptable error rate of 2.0% and confidence level of 95.0%. To compare the proportions, the chi-square test or Fisher’s exact test was applied. The association between scoliosis and risk factors was evaluated by logistic regression, being significant *p* <0.05.

**Results:**

The prevalence of scoliosis was 24.3%, higher in obese patients and students who adopted a sitting position for a long period of time. Obese students showed a 1.8 times higher chance of testing positive Adam’s Forward Bend Test when compared to normal-weight/lean and 2.1 times higher chance compared to overweight students. The sitting position for watching television increases the chance of testing positive Adam’s test in 38.0%, when compared to the lying position. Obesity increases the risk of testing positive Adam’s test in 74.0 and 98.0%, when compared, respectively, to the underweight/normal weight and overweight.

**Conclusions:**

There was a high prevalence of scoliosis in students from public elementary schools in Santos. The most influential factors for this deviation of the spine were obesity and the position adopted by students to watch television.

## RESUMO

**Objetivo:**

Avaliar a prevalência de escoliose e os fatores de risco em alunos do ensino fundamental.

**Métodos:**

Estudo transversal realizado em 954 alunos em 2015. O instrumento envolvido foi um questionário sobre hábitos posturais, condições socioeconômicas e fatores demográficos. As medidas antropométricas, incluindo altura e peso, a inspeção visual da deformidade da coluna vertebral, o nivelamento dos ombros e o Teste de Adams foram obtidos. A amostra foi calculada na frequência esperada de 12,3%, erro aceitável de 2,0% e nível de confiança de 95,0%. Para comparar as proporções, o teste do qui-quadrado ou o teste exato de Fisher foram aplicados. A associação entre escoliose e fatores de risco foi avaliada por regressão logística, sendo significante *p*<0,05.

**Resultados:**

A prevalência de escoliose foi de 24,3%, superior em obesos e estudantes que adotavam posição sentada durante um longo período de tempo. Estudantes obesos mostraram risco 1,8 vez superior no Teste de Adams positivo quando comparados aos eutróficos/magros e risco 2,1 vezes superior comparados aos alunos com sobrepeso. A posição sentada para assistir à televisão aumenta a chance de Teste de Adams positivo em 38,0% quando comparada à posição deitada. A obesidade aumenta o risco de Teste de Adams positivo em 74,0 e 98,0% quando comparada, respectivamente, à magreza/eutrofia e ao sobrepeso.

**Conclusões:**

Houve elevada prevalência de escoliose em estudantes de escolas públicas do ensino fundamental em Santos. Os fatores que mais influenciaram esse desvio de coluna foram a obesidade e a posição adotada pelos alunos para assistir à televisão.

## INTRODUCTION

The postural problems of the spinal column, mainly scoliosis, have been considered a serious issue of public health, because they have a high incidence in the workforce, disabling it temporarily or permanently for professional activities.^1^

Scoliosis is a severe postural alteration, characterized as a lateral deviation accompanied by the distortion of individual parts of the spinal column, which makes it a morphological deformation,^2^ thus causing emotional problems related to visually impaired esthetics, pain, as well as pulmonary mechanic-related problems.^3^

These changes, in general, are developed in childhood and adolescence and, when ignored, may progress and become irreversible. Knoplich,^4^ in 1985, observed that incorrect postural habits adopted since elementary education are alarming, as skeletons of children are in growth phase and muscle and skeletal structures present greater tolerability to loads and are more susceptible to deformations. The prevalence of scoliosis varies in different regions of Brazil and throughout the world, oscillating between 1.0 and 15.8% in children and adolescents.^5^ ^,^ ^6^ ^,^ ^7^ ^,^ ^8^ ^,^ ^9^ ^,^ ^10^

According to Stokes and Moreland,^11^ in 1987, Adam’s Forward Bend Test was recommended for detection of scoliosis as it accentuates the deformity on the surface of the torso, being widely used throughout the world as the basis for this problem’s evaluation in schools.^7^ ^,^ ^8^ ^,^ ^10^ ^,^ ^11^ According to Santos et al.,^12^ students have minimal knowledge about the consequences of spinal deviations and about the possible causes that generate them, because they do not receive information or guidance about postural habits and disorders related to posture. A large part of school-age children remain seated for hours in an improper manner and make use of improper securities, both in schools and at home.

In this context, the objective of this study was to estimate the prevalence of scoliosis in children from 1st to 4th grades of elementary school, enrolled in the municipal schools of the city of Santos, and verify its association with postural habits adopted in their daily activities and with demographic, socioeconomic, and anthropometric variables.

## METHOD

A cross-sectional study was carried out in the year 2015, when 954 questionnaires were applied to families and children. Simultaneously, Adam’s test was applied to detect the presence of scoliosis, and the height and weight of the students were also measured to calculate the body mass index (BMI), in addition to the inspection of spinal deviations and the leveling of shoulders in children from 1st to 4th grades of elementary school, who were enrolled in municipal schools of Santos.

We used the Epi Info program version 6 (November 1996). The sample was calculated with an expected frequency of 12.3% (based on the study of Saints et al.,^12^ in Cuiabá, Mato Grosso, because it is a representative sample and it uses the Adam’s Test), an acceptable error of 2.0% and confidence level of 95.0%. The calculation was made, taking into account 12,129 students enrolled in the municipal schools of Santos. The total sample was composed of 954 students, or 10 students from each of the 7 grades of 15 randomly selected schools. The randomly selected students were invited to join the study, and the presence of their caregivers was required. After the approval by the Committee for Ethics in Research on Human Beings of the University Center Lusíada de Santos, with the authorization of the Municipal Secretariat of Health and Education of Santos, the project was presented to the directors, coordinators, and teachers of the randomly selected schools. The informed consent form about the research, was presented to the caregivers, and, after approval, the questionnaire administration and the physical examination were started.

In the first stage, we administrated the questionnaire with demographic and socioeconomic data and postural habits. In the second stage, the physical examination was done in a closed room on each student (accompanied by the caregivers) by two observers and, in case of doubt, by a third observer. The students were asked to remain without shirts and barefoot. The physical examination consisted in: measurement of height and weight in order to calculate the BMI; inspection of visible spine deviations; leveling of the shoulders, putting the student against the wall (with a line), front and back; and application of the Adam’s test. For its implementation, the students were requested to remain in orthostatic position, barefoot, with feet together, knees straight, and bent forward until their backs were in a horizontal position, keeping the arms hanging, the palms of the hands together, with fingers in opposition. The test was considered positive if there was a vertebral rotation.

A descriptive analysis was performed, with presentation of tables of frequencies for categorical variables. Chi-squareor Fisher’s exact tests were applied to compare proportions, and logistic regression analysis was used to study associated factors to the positive Adam’s test. The level of significance adopted for the statistical tests was 5.0%. All statustical tests were applied by SAS *System for Windows (Statistical Analysis System)*, version 9.4 (SAS Institute Inc, 2002-2012, Cary, NC, USA).

## RESULTS

The overall prevalence of scoliosis was 24.3%. In the analyses performed, significant associations were found between posture while watching TV, nutritional evaluation, and inspection of the vertebral column. Students who have the habit of watching television in the sitting position had higher percentages of positive Adam’s test, compared to those who watch TV in the lying position. Students who had their spines inspected and were found without deviations, had higher percentages of negative Adam’s test, as shown in Tables 1 and 2.

**Table 1:**
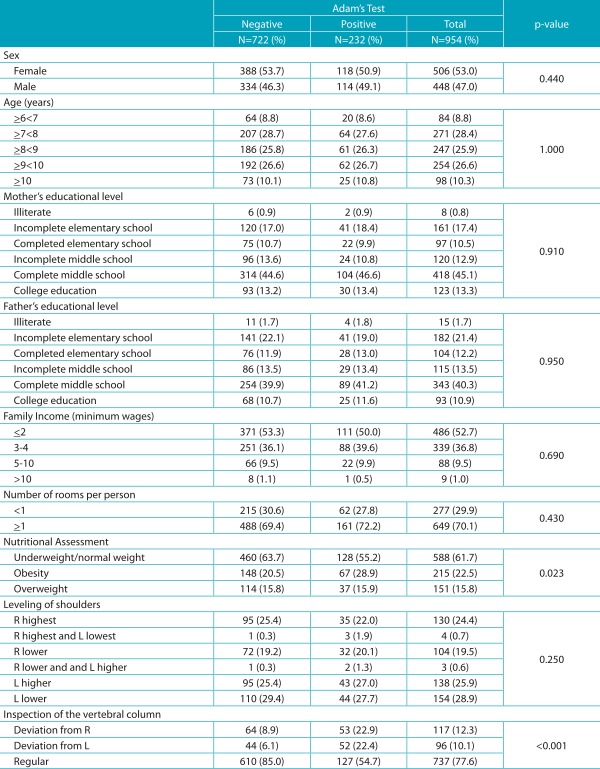
Prevalence of positive Adam’s test according to demographic, socioeconomic, and anthropometric variables.

R: right; L: left.

**Table 2:**
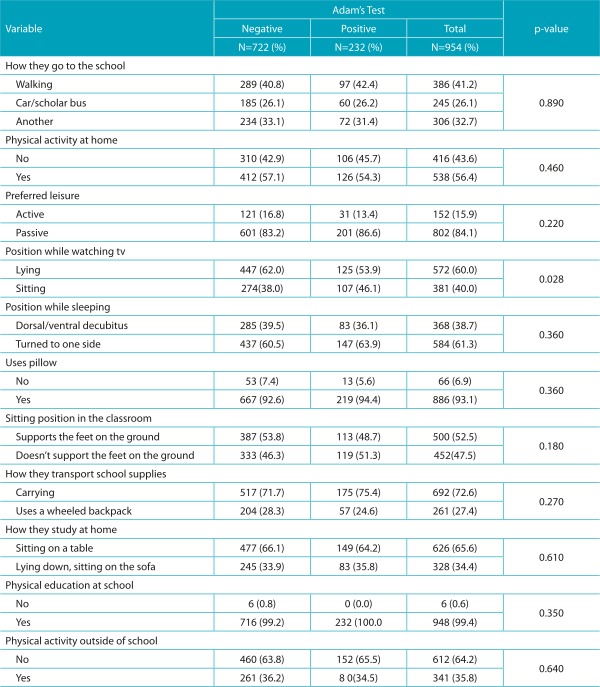
Prevalence of positive Adam’s test according to postural habits adopted in daily activities.

The results of the analysis of univariate and multiple logistic regressions to identify associated factors to positive Adam’s test, in the model with a small number of observations due to missing data on some variables, obese patients presented 1.8 times higher chances of having a positive Adam’s test, when compared to normal-weight or underweight, and 2.1 times higher chances when compared to overweight patients. Excluding the variables with greater frequency of missing data, the sitting position while watching TV increased the chance of positive Adam’s test in 38.0% when compared to the lying position, while obesity increased the risk of positive Adam’s Test in 74.0%, compared to underweight/normal weight, and in 98.0%, when compared to overweight, as shown in Tables 3 and 4.

**Table 3:**
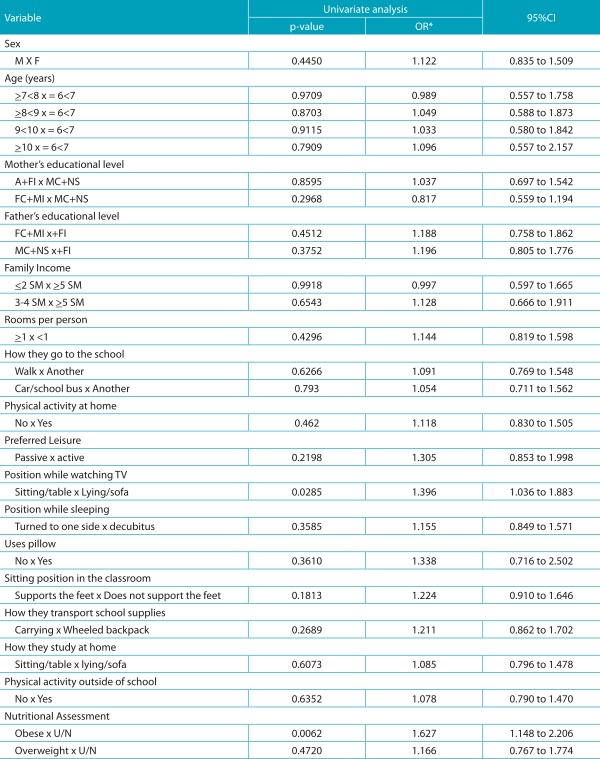
Univariate logistic regression analysis to identify associated factors to positive Adam’s testing.

*OR: odds ratio; CI95% confidence interval for OR; M: male, F: female; A: illiterate; FI: incomplete elementary school; FC: completed elementary school; MI: incomplete middle school; MC: completed medium complete; NS: college education; SM: minimum wage; M/E: underweight+ normal weight.

**Table 4:**
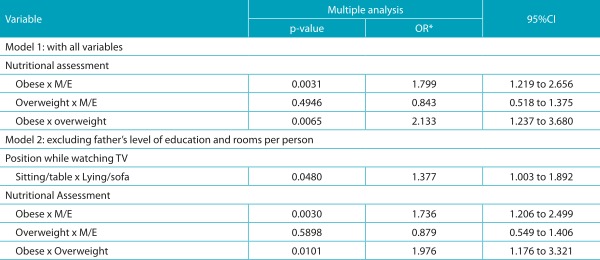
Multiple logistic regression analysis to identify associated factors to positive Adam’s Test.

*OR: odds ratio; IC95%: 95% confidence interval for OR; M/E: underweight + normal weight.

## DISCUSSION

The prevalence of scoliosis in 24.3% of the sample was superior to the results found in most of the literature. It was far superior to that found by Nery et al.,^8^ in 2010, of 1.4% in adolescent students between 10 and 14 years in the municipality of Carlos Barbosa, Rio Grande do Sul; and also to the study of Holy et al.^7^, in 2002, of 12.3% in municipal schools of Cuiabá, Mato Grosso, and the observations made by Souza et al.,^13^ in 2013, of 4.3% in adolescent students from public schools in Goiânia, Goiás. However, the prevalence of scoliosis was lower than the one reported by Vieira et al.,^14^ in 2011, of 26.3% in the age group of preschoolers in Londrina, Paraná, thereby stressing the importance of an early diagnosis for the implementation of preventive measures. There is also a variation of the prevalence in other countries. In 2014, Ortega et al.^15^ ^,^ ^16^ found a prevalence of 14.2% of scoliosis in Mexican school children with an average age of 10 years, and of 36.3% in Spanish students with an average age of 8.5 years, while Minghelli et al.,^17^ also in 2014, found 4.2% of adolescents between 10 and 16 years with scoliosis in southern Portugal. The variation found in the prevalence of the various studies may be due to the different methods employed to detect scoliosis, i.e., it is more difficult to compare results because there is no standardization in the methodology of the surveys. Another possible explanation to be given to this variation is the number of differences found in the age groups studied. Second Braccialli and Vilarta,^1^ the period of pubertal growth spurt is associated with the development and acceleration of postural deviations. However, what has occurred in the present study was a higher prevalence in the age groups from 6 to 10 years, than in assessments of adolescents,^8^ ^,^ ^13^ ^,^ ^17^ suggesting that other factors may be involved. In this context, this study has limitations because it does not include several factors that may be associated with postural deviations, such as the adequacy of the sofa or chair used for study or for leisure, the weight of the backpack with school supplies, and the period in which it is charged.^8^

There was no statistical difference in the prevalence of scoliosis between genders. However, a variation in the results is found in literature. Tavares et al.^18^ did not find also such association; however, in the study of Fields et al.,^19^ there was a predominance of scoliosis in females, while Ferriani et al.^20^ showed a higher frequency in males. It is possible that the explanation for this variation is that, in the age groups studied, the majority of girls has not yet reached the peak of growth rate, which occurs in a period prior to the boys, and higher prevalence of postural deviations in the periods of growth spurts is expected.^1^

No association was found between the scoliosis and the majority of demographic and socioeconomic variables studied, as there was also none between the posture adopted in daily activities, with the exception of the sitting position while watching TV. There is a large variation of these variables in the analyses found in literature, and the importance of posture in its execution is mentioned by the majority of studies.^8^ ^,^ ^15^ ^,^ ^21^ The students in growth phase and with sitting postures unsuitable for the performance of their tasks in the classroom or at home can acquire postural deviations. Ortega et al.^16^ report that adopting an improper sitting posture increases the likelihood of spinal deviation. Braccialli and Vilarta^1^ reported that staying in the sitting position for long periods of time, maintaining a static posture, without movement and, sometimes inadequate movement, results in overload of the vertebral column, which supports the body. The dorsal decubitus position would reduce the load over lumbar region compared to sitting. The habit of watching television, which is a part of everyday life of children is added to the likelihood of postural deviations. Penha et al.^6^ mention that adopting a improper posture is related to a defective relationship between the various parts of the body, which produces greater tension in the muscle structures, causing imbalance in the body. It is also important to analyze, from the point of view of ergonomics, the appropriateness of the chair or sofa to the height of the child who is sitting watching television, the slope of the chair and, also, the wrong postures of sitting by tilting the shoulders forward. The adoption of a improper sitting posture during the growth phase due to the use of a inadequate piece of furniture can facilitate spinal deviations.^1^ ^,^ ^6^

The association between positive Adam’s test and the obesity as found in this study, is not observed in several studies in the literature, such as Nery et al.,^8^ Jannini et al.,^22^ Pinto et al.,^23^ and Souza Junior et al.^24^ However, in 2011 Silva et al.^25^ found a higher prevalence of postural deviations in obese children and adolescents. Postural deviations, in an attempt to adjust the posture, occur in overweight children, in order to avoid localized muscular fatigue.^23^ ^,^ ^25^ These diverging data continue to suggest that other factors not addressed in this study may also influence the presence of scoliosis, as the excess weight of school supplies^8^ and the ergonomics in school and at home.^1^ ^,^ ^6^

Several studies show that the increase of knowledge and learning of healthy postural habits has a positive effect.^26^ ^,^ ^27^ ^,^ ^28^ ^,^ ^29^ In this study, however, the knowledge of teachers, parents, and students about postural habits was not discussed. Even in pediatric consultations it is known that it is unusual to evaluate the posture of the child or adolescent, making it difficult to ascertain the effect of knowledge of postural habits of the patient. With this, the importance of the school in a preventive proposal toward the orientation of proper postural habits, especially in this growth phase, is considered critical for the acquisition of postural deviations, and is emphasized on.

There was a high prevalence of scoliosis in students from elementary public schools in Santos. The factors that most influenced this spinal deviation were obesity, and the position adopted by the students to watch television.
